# Do the Fertility Drugs Increase the Risk of Cancer? A Review Study

**DOI:** 10.3389/fendo.2019.00313

**Published:** 2019-05-24

**Authors:** Zohre Momenimovahed, Safoura Taheri, Azita Tiznobaik, Hamid Salehiniya

**Affiliations:** ^1^Department of Midwifery and Reproductive Health, School of Nursing and Midwifery, Qom University of Medical Sciences, Qom, Iran; ^2^Department of Midwifery and Reproductive Health, School of Nursing and Midwifery, Tehran University of Medical Sciences, Tehran, Iran; ^3^Department of Midwifery and Reproductive Health, School of Nursing and Midwifery, Ilam University of Medical Sciences, Ilam, Iran; ^4^Department of Midwifery and Reproductive Health, School of Nursing and Midwifery, Hamedan University of Medical Sciences, Hamedan, Iran; ^5^Social Determinants of Health Research Center, Birjand University of Medical Sciences, Birjand, Iran; ^6^Department of Epidemiology and Biostatistics, School of Public Health, Tehran University of Medical Sciences, Tehran, Iran

**Keywords:** infertility, ovulation induction, cancer, infertility treatment, controlled ovarian stimulation, fertility agents, neoplasms

## Abstract

**Aim:** All over the world, many couples cannot conceive a child and have problems with pregnancy. Ovulation-inducing drugs are among the most important drugs used for the treatment of infertility. In recent years, there have been many debates about the relationship between fertility medication and cancer. Due to the lack of comprehensive study of this matter, and as understanding the relationship between the use of fertility drugs and cancer is of importance, the present study was conducted to investigate the relationship between infertility drugs and cancer in women.

**Materials and Methods:** To determine the relationship between infertility treatment and cancer, a comprehensive search was carried out in databases such as; Medline, Web of Science Core Collection, and Scopus using keywords words; “infertility,” “ovulation induction,” “cancer,” “infertility treatment,” “ART,” “tumor,” “controlled ovarian stimulation,” “fertility agents,” and “neoplasms.” Full-text, English language, and original articles were included in this study.

**Results:** In total, 81 articles were entered into the study. The relationship between fertility medications and breast, ovary, endometrial, uterus, colon, thyroid, skin, cervical, and non-Hodgkin's lymphoma cancers were studied. Although the relationship between fertility medications and cancer is theoretically justifiable, most studies have shown that risk of cancer will not increase after fertility treatment.

**Conclusion:** The results of this study did not show that fertility medications increase the risk of cancer among users. In summary, the relationship between infertility treatment and cancer incidence remains an open question.

## Introduction

All over the world, 48.5 million couples have problems with pregnancy, and many children are born with the help of fertility treatments, which primarily occurs in developing countries (1). After cancer and cardiovascular disease, infertility is the third most common disease (2). With increasing numbers of couples delaying parenting attempts, the prevalence of infertility is on the rise. Decreased fatality is one of the most important issues in Western countries, and is an essential part of reproductive health (1), which affects different aspects of life and imposes huge economic burdens on societies (2). Infertility is affected by various physiological, genetic, environmental, social, infectious, and nutritional factors (1, 3). Assisted reproductive technology (ART) is one of the most important strategies used today to increase the chance of fertility among infertile people (4). The ART uses many medications and techniques to increase the chance of fertility (5). Ovulation-inducing drugs are among the most important drugs used for the treatment of infertility, which affect the ovaries by increasing the levels of estrogen, progesterone, and gonadotropins. Complications of these drugs include ovarian hyper-stimulation syndrome, osteoporosis, and adverse pregnancy outcomes (6, 7).

In recent years, there have been many debates about the relationship between ovulation-inducing drugs, infertility treatment, and cancer (8–10). Changes in endogenous hormones that occur following the use of these drugs have raised many concerns about the safety of these treatments. Due to increasing infertility and the subsequent increase in the use of fertility drugs in recent years, investigating the long-term effects of these drugs are considerably important. It also raises the following question: Can the use of fertility drugs in the long-term cause cancer? Since understanding the relationship between the use of fertility drugs and cancer is important and due to the lack of comprehensive study in this regard, the present study was conducted to investigate the relationship between infertility drugs and cancer among women.

## Materials and Methods

### Search Strategy

To determine the relationship between infertility treatment and cancer, a comprehensive search for reliable articles was carried out in databases such as; Medline, Web of Science Core Collection (Indexes = SCI-EXPANDED, SSCI, A & HCI Timespan), and Scopus (all years), using keywords, including “infertility,” “ovulation induction,” “cancer,” “infertility treatment,” “ART,” “tumor,” “controlled ovarian stimulation,” “fertility agents,” and “neoplasms.” Combinations of these keywords were also used for the search. All keywords were checked with PubMed Medical Subject Heading (MeSH). Then, a manual search was conducted in valid journals for full-text articles and related systematic reviews. All retrieved articles were entered into the Endnote X7 software in one database. In order to reduce errors during the review phase, the PRISMA statement and Moher et al. (11) guidance were used.

### Inclusion Criteria

Two researchers carefully reviewed the retrieved articles. The criteria for entering the study included; full-text articles, English language and original articles (case-control, retrospective and prospective cohort), the use of keywords in the title or abstract, articles with an abstract, and articles that have reviewed a type of malignancies associated with the use of fertility drugs. *In vitro* fertilization (IVF) and each of the clomiphene citrate regimens, the gonadotropins, human chorionic gonadotropin (hCG), human menopausal gonadotropin (hMG), gonadotropin-releasing hormone (GnRH) agonist, and antagonists were studied alone or in combination.

### Exclusion Criteria

Case reports, case series, systematic reviews, meta- analysis, and animal studies were excluded.

### Extracting and Analyzing the Data

The articles were categorized according to their specific characteristics including the number of participants, type of study, and type of fertility regimen. The risk scale in this study included; Standardized Incidence Ratio (SIR), Incidence Rate Ratio (IRR), Hazard Ratio (HR), and Odds Ratio (OR).

## Results

### Characteristics of the Selected Studies

**Figure 1:**
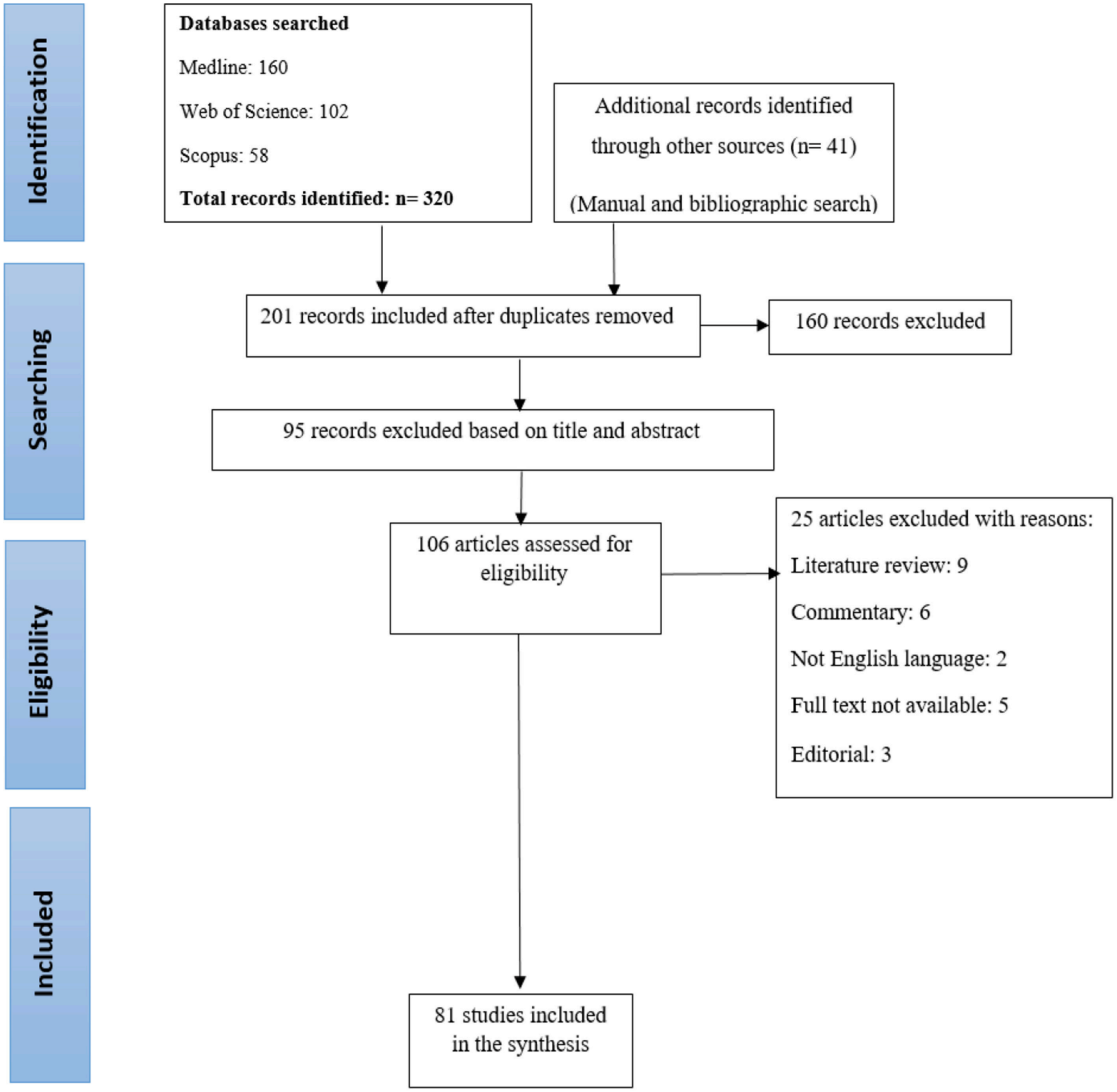
Flowchart of the included eligible studies in review.

After a comprehensive search through the databases, 320 articles were entered into the study and the references of 41 articles were manually reviewed. Duplicate articles were removed using Endnote software (*n* = 160). During the initial evaluation, 201 articles were selected for the review. After reviewing the title and abstract, 95 articles that were not consistent with the purpose of this study or did not meet the inclusion criteria were removed. The full texts of other articles were carefully examined by two researchers, and 25 articles were removed for scientific reasons (literature review: 9, commentary: 6, not English language: 2, full text not available: 5, editorial: 3). At the end, 81 articles were selected for the review (Figure 1).

### Relationship Between Steroid Hormones and Cancer

In general, cancer occurs as a result of the division of mutated cells. Therefore, the hormone and any factor that stimulates the growth of the cells can affect the occurrence of cancer (12). The relationship between steroid hormones and some types of cancers, such as breast and endometrium cancers, is well-documented (12–14). Estrogen, progesterone, and testosterone are steroid hormones that are produced following a series of biological responses and are derived from cholesterol. Estrogen, which is one of the most important female sex hormones, is produced in the ovaries, the adrenal cortex, and the placenta, and leads to the growth of the reproductive system and the development of sexual traits (15). Based on the results of studies, free estradiol and albumin-bounded estradiol are associated with an increased risk of breast cancer (16) and can enhance the progression of metastatic breast cancer (17). High levels of estradiol and estrogen hormones in women with breast cancer contribute to the progression of metastatic breast cancer (18). Estrogen can contribute to the onset of cancer by affecting the mitosis duplication of epithelial cells. Increasing mitotic activity can play a role in increased probability of mutation and DNA damage, and reduced apoptosis and growth of early tumors (13, 19).

The role of gonadotropins in the occurrence of cancer has also been discussed by researchers. High levels of LH and FSH cause exposure to uncontested estrogen in the menstrual cycle, which contributes to an increase in cancer. On the other hand, stimulating the synthesis of mitogenic growth factors by gonadotropins can contribute to the growth of breast cancer cells (20, 21). In addition, high levels of gonadotrophin, predict a weaker prognosis in people with breast cancer (21).

### Analysis Based on the Type of Cancer (Table 1)

#### Breast Cancer

Breast cancer is a multifactorial disease and several risk factors are involved in its onset. The majority of breast cancers are hormone dependent (22). Several studies have pointed to the etiologic role of endogenous and exogenous hormones in increasing the risk of breast cancer. Therefore, the possible relationship between ovulation-inducing drugs and increased risk of breast cancer has been the subject of discussions by researchers (23–25). Compared with normal ovulation cycle, oestradiol concentration increase up to 10-fold in ovulation stimulation cycle (26). Treatment with fertility drugs is associated with an increased risk of breast cancer diagnosis in the first year after the treatment (27). The result of a cohort study of 808,834 women showed that the risk of breast cancer increased in individuals who gave birth following ART [adjusted hazard risk (HR) 1.20, 95% CI 1.01–1.42]. However, in this study confounding factors such as age of menarche, family history, history of breastfeeding, and obesity were not controlled (28). In a cohort study, the risk of breast cancer was increased among individuals who used ovulation-inducting drugs, although this risk was not statistically significant in primiparous women (29). The result of a case-control study of 35–64 years old women showed that, although the use of fertility drugs was not associated with an increased risk of breast cancer in general, the relative risk of breast cancer in people with a hMG use for more than 6 months or 6 cycles was about 2.7–3.8 (30). A case-control study found that IVF treatment after the age of 30 years was associated with an increased risk of breast cancer, however, the characteristics of breast tumors in subjects treated with IVF did not differ with the general population (31). Although the results of the above studies suggest a relationship between infertility treatment and the risk of breast cancer, a large, population based cohort study between 1991 and 2010 stated, regardless of infertility treatment, the risk of breast cancer in people who are incapable of conceiving or maintaining fetuses is increased by 2 times (32). Meanwhile, many researchers could not show a positive relationship between fertility treatment and breast cancer (32–38). The result of a cohort study of 98,997 women aged 40–65 years showed no relationship between the risk of breast cancer and infertility treatment, the type of treatment, the type of drug, the age of the person at the start of treatment, and the duration of treatment. The author of this study suggested that infertility treatments may be associated with an increased risk of breast cancer among women with a family history of breast cancer (39). In a cohort study of 54,362 women, Jensen showed no relationship between the duration of fertility drugs use, the time since the first use of drugs, and the risk of breast cancer (40). The results of a cohort study pointed to the potential association between family history and the use of ovulation-inducing drugs in the incidence of breast cancer (39). In general, according to the results of related studies, the relationship between fertility treatment and breast cancer has not been proven.

**Table 1:** Characteristic study of effects of fertility drugs on cancer.

**References**	**Country**	**Design**	**Period**	**Study** ** population**	**Mean age at** ** entry (Year)**	**Mean follow** ** up (Year)**	**Adjusting** ** factor(s)**	**Type of infertility treatment**	**Type of malignancy**	**Number of stimulated cycles**	**Effect estimates**	**Main finding(s)**
Reigstad et al. (28)	Norway	Cohort	1984–2010	Total:808,834 Case: 16,626 Control: 792,208	Age range: Case: 29.7–35.3 Control: 22.9–29.9	16.0 (12,401,121) person- years	Age Parity Age at first birth Region of residence	IVF ICSI	Breast		HR	Increased risk of breast cancer in women with ART.
Burkman et al. (30)	US	Case-Control	1994–1998	Case: 4,575 Control: 4,682	Age range: 35–64		Age Race Strata of study center	Clomiphene citrate hMG	Breast	<6-≥6	OR	Increased risk of ductal breast cancer in women who use hMG.
Williams et al. (32)	UK	Cohort	1991–2002	Total:255,786	34.5	8.8 (2,257,789 person- years)			Breast Corpus uteri Ovary	1.8	SIR	No association between ART and corpus uteri and invasive breast cancer risk. Increased risks of *in situ* breast cancer and invasive and borderline ovarian tumors in women with ART.
Dor et al. (23)	Israel	Cohort	1981–1992	Total:5,026	34.0	3.6 (18,291 person- years)		Clomiphene citrate hMG GnRH analogs	Breast Ovary Cervix Endometrium	1-≥6	SIR	No association between fertility treatment and cancer risk.
Luke et al. (33)	US	Cohort	2004–2009	Total:113,226 Case: Control: 53,872	Case: 37.8 Control:35.3	4.8 (263,457 person-years)	Age at cycle start Parity Infertility diagnosis Number of ART cycles Cumulative FSH dosage ART outcome	FSH clomiphene citrate	Endocrine Melanoma Breast Ovary Uterine Female genital	1, 2, 3, 4, or ≥5	SIR HR	No association between fertility treatment and cancer risk.
Gauthier et al. (39)	France	Cohort	1990–2000	Total:92,555 Case: 6,602 Control:	Age range: 40–65	9.7	Education Smoking BMI Self and familial history of breast cancer Age at menarche Menopausal status Parity Age at first full term pregnancy	Clomiphene citrate Gonadotropins	Breast	Mean: 13 months	RR	No association between fertility treatment and breast cancer risk.
Jensen et al. (40)	Denmark	Cohort	1965- 1998	Total:54,379		8.8	Number of childbirth	FSHhCGhMGGnRH	Breast		RR	No association between fertility treatment and breast cancer risk.
Sanner et al. (47)	Swedan	Cohort	1961–2004	Total:2,768 Case: 1,153 Control:1,615	Age range: 16–45	33.0	Indication of treatment Age OCP Parity Pelvic surgery before the infertility treatment Pregnancy with completed birth in the follow-up period	Clomiphene citrate Gonadotropins	Ovary	1–4 cycles	SIR RR	No association between hormonal infertility treatment and invasive ovarian epithelial cancer.
Silva et al. (48)	London	Cohort	1963–1999	Case:7,425 Control: 1,727	28.1	21.4	Age Parity	Clomiphene citrate Gonadotropinss	Breast Uterine Ovary	2–3 cycles	Period-age-standardized mortality (SMR) SIR	No association between ovulation stimulation drugs and cancer risk.
Potashnik et al. (49)	Israel	Cohort	1960–1984	Total:1,197 Case: 780 Control: 417	27.5	17.9 (21,407 person-years)		Clomiphene citrate hMG	Breast Ovary Uterine Cervix		SIR	No association between fertility drugs and cancer risk.
Venn et al. (50)	Australia	Cohort	1978–1992	Total:10,358 Case: 5,564 Control: 4,794	Case: 32 Control: 31	Case: 5.2 Control: 7.6 (31,272 person-yers)	Age Infertility type	IVF	Breast Ovary	Median: 2 cycles	SIR	No association between IVF and cancer risk.
Franceschi et al. (51)	Italy	Case-Control	1992	Case: 195 Control: 1,339	Case: 55 Contol: 56				Ovary		OR	No association between ovulation stimulation drugs and cancer risk.
Modan et al. (85)	Israel	Cohort	1964–1974	Total:2,496	28.7	21.4 (54,413 person- years)		Clomiphene citrate hMG	Ovary Endometrium Breast Melanoma Thyroid		SIR	No association between ovulation stimulation drugs and ovarian cancer risk.
Trabert et al. (53)	US	Cohort	1965–1988	Total:9,825	30.1	17.6 (256,448 person- years)	Study site Age Gravidity	Clomiphene citrate gonadotropins	Ovary	≥6	RR	No association between ovulation stimulation drugs and cancer risk. (exception: Use of Clomiphene citrate and failure to pregnancy was related to ovarian cancer risk)
Jensen et al. (54)	Denmark	Cohort	1963– 1998	Total:54,362	30	16.0 (957,454 person-years)	Parity	Clomiphene citrate Gonadotropins hCG GnRH	Ovary	1-≥10	RR	No association between fertility drugs and cancer risk.
Doyle (86)	UK	Cohort	1975–1989	Total:5,556		43,811 person-years	Age Calendar year Parity following the last treatment cycle		Breast Uterine Ovary		SIR	No association between fertility drugs and cancer risk.
Stewart et al. (87)	Australia	Cohort	1982–2002	Total:21,639 Case: 7,544 Control: 14,095	31.2	16.5 (365,775 person- years)	Age Socioeconomic status	IVF	Ovary		HR	Increased risk of borderline ovarian tumors in women with IVF
Mosgaard et al. (58)	Denmark	Case-Control	1989–1994	Case: 231 Control: 1,721	Case: 43.6 Control: 46.0		Age Region of residence Use of oral contraceptives Use of hormone replacement therapy Smoking	Clomiphene citrate hCG hMG	Ovary		OR	No association between fertility drugs and borderline ovarian cancer risk.
Bjørnholt et al. (59)	Denmark	Cohort	1963–2006	Total:96,545	30.3	11.3 (1,222,252 person- years)	Parity Cause of infertility	hCG clomiphene citrate Gonadotropins GnRH analogs	Ovary	1-≥4	RR	No association between fertility drugs and borderline ovarian cancer risk.
Liat et al. (24)	Israel	Cohort	1964–1974	Total:2,431	28.6	33.8 (88,186 person- years)		Clomiphene citrate hMG	Ovary Breast Endometrium		SIR	No association between fertility drugs and borderline ovarian cancer risk. Uncertain association between fertility drugs and breast and endometrial cancer risk.
Althuis et al. (69)	US	Cohort	1965–1988	Total:8,431	30	18.8 (155,658 person- years)	Calendar year Age Study site	clomiphene citrate Gonadotropins	Uterine	<6-≥6	RR	Increased risk of uterine cancer in women who use clomiphene citrate
Jensen et al. (70)	Denmark	Cohort	1965–1998	Total:54362	30	16.0 (957,887 person- years)	Parity	Clomiphene citrate Gonadotropins hCG GnRH	Uterine	1-≥10	RR	Increased risk of uterine cancer in women who use gonadotropins, clomiphene and human chorionic gonadotropin
Hannibal et al. (88)	Denmark	Cohort	1963– 1998	Total:54,362	30	8.8	Age at first live birth Use of OCP Parity	Clomiphene citrate Gonadotropins hCG GnRH	Thyroid	1-≥6	RR	Increased risk of thyroid cancer in women who use clomiphene
Calderon-Margalit. (29)	Israel	Cohort	1974–2004	Total:14,463	Case: 28.1 Control:27.5	29.0 (424,193 person- years)	Age Socioeconomic status Mother's geographic origin Body mass index Parity	Clomiphene citrate	Breast Uterus Ovary Cervix Non-Hodgkin lymphoma Malignant melanoma Thyroid Colon		HR	No association between fertility drugs and ovarian cancer risk. Increased risk of uterine and borderline breast cancer, malignant melanoma and non-Hodgkin lymphoma in women who use ovulation induction
Kallen et al. (84)	Swedan	Case-Control	1982–2006	Case:24,058 Control: 1,394,061	32.0		Year of delivery Maternal age Parity Smoking	IVF	Breast Cervix Ovary Placenta CNS Malignant melanoma Thyroid Colon		OR	Decreased risk of breast and cervical cancer in women with IVF
Leeuwen et al. (63)	Netherlands	Cohort	1983–2007	Case: 19,146 Control:6,006	At end of follow up Case:47.5 Control:49.4	14.7	Age Parity Subfertility cause	IVF	Ovary	1-≥5	SIR	Increased risk of borderline ovarian cancer in women with IVF
Orgéas et al. (89)	Sweden	Cohort	1961–2004	Total:1,135	27	35,092 person- years	Age Calendar period of breast cancer diagnosis Age at first birth Parity	Clomiphene citrate Gonadotropins	Breast	1-≥4	SIR	No association between fertility drugs and breast cancer risk.
Lundburg et al. (90)	Sweden	Cohort	1982–2012	Total:1,340,211		Case: 9.6 Control: 14.6	Age Parity Calendar time Educational level Country of birth Family history of breast cancer Age at first birth	Clomiphene citrate Gonadotropins	Breast		HR	No association between ovarian stimulation and breast cancer risk.
Kessous et al. (46)	Israel	Cohort	1988–2013	Total:10,6031	Case: 30.4 Control: 28.3	11.6	Maternal age Obesity		Ovary Uterine Cervix Breast		HR	Increased risk of ovarian and uterine cancer in women who used fertility treatment
Kristiansson et al. (91)	Sweden	Cohort	1981–2001	Case: 8,716 Control: 64,0059	Age range:21–43	Case: 6.2 Control: 7.8	Age at follow-up Calendar year at follow-up Number of siblings and multiple births Age at first conception	IVF	Ovary Uterine Cervix Breast		RR	decreased incidence of carcinoma *in situ* of the cervix and breast cancer in women who used fertility treatment
Pappo et al. (92)	Israel	Cohort	1986–2003	Total:3,375	32.1	8.1 (27,327 person-years)	Age Continent of birth		Breast	1–18	SIR	Increased risk of breast cancer in women who used fertility treatment
Spaan et al. (81)	Netherlands	Cohort	1989–2013	Case: 19,157 Control:5,950		Case: 20.7 Control: 23.5	Age Parity IVF cycles	IVF	Colon	1-≥7	SIR	No association between IVF and colorectal cancer risk.

#### Ovarian Cancer

Ovarian cancer is a rare and the most fatal gynecological disease worldwide (41). Regardless of infertility treatments, the risk of ovarian cancer may be altered with nulliparity (24), and infertility (42, 43). Therefore, the relationship between infertility treatment and ovarian cancer is difficult to prove. The “incessant ovulation theory” states that uninterrupted ovulation can contribute to the development of ovarian cancer by damaging ovary epithelium and, therefore, any factor that contributes to the reduction of ovulation can have a protective effect against ovarian cancer (44). Many studies have indicated that an increased risk of ovarian cancer is associated with the intake of clomiphene citrate and gonadotropin (25, 45). A cohort study conducted from 1988 to 2013 revealed that, the risk of ovarian cancer is increased in individuals treated with IVF (adjusted HR 3.9; 95% CI 1.2–12.6), (46). The results of a cohort study showed an increase in the incident of ovarian cancer after exposure to clomiphene citrate. The results also indicated that risk of cancer increases with increasing dosage of the drug among nulliparous women (25). Use of clomiphene citrate, due to ovulation disorders, increases the risk of ovarian cancer (SIR = 7.47; 95% CI 1.54–21.83), (47). Although a number of studies have suggested that using ovulation-inducing drugs is associated with ovarian cancer, many of them have not shown any significant increase in the risk of ovarian cancer by taking ovulation-inducing drugs (29, 33, 48–52). The result of a retrospective cohort study showed that women who use clomiphene citrate and remained nulligravid are more likely to develop ovarian cancer than those who use this drug and get pregnant (RR 3.63, 95% CI 1.36–9.72 vs. RR 0.88, 95% CI 0.47–1.63), (53). In a cohort study of 54,362 women, authors reported that the risk of ovarian cancer does not increase with the use of clomiphene citrate, gonadotropins, human chorionic gonadotropin, and gonadotropin-releasing hormone, and that there is no relationship between the duration of drug use, duration of follow-up, and pregnancy (54). An increased risk of ovarian cancer among those taking ovulation-inducing drugs should be an issue of interest to therapists and, therefore, more attention should be paid to the people's choices (45).

In several studies, researchers have investigated the relationship between borderline ovarian tumors and fertility treatments. The risk to develop borderline ovarian tumors increased in women undergoing IVF, while childbirth, hysterectomy and sterilization do not have protective effects (55). In a cohort study, the use of clomiphene citrate and gonadotropins increased the risk of borderline ovarian tumors by up to 3 times (SIR = 3.61; 95% CI 1.45–7.44), (47). A case-control study showed a relationship between borderline ovarian tumors and the use of ovulation-inducing drugs, especially hMG (56). The result of a study revealed that, although treatment with clomiphene citrate for <1 year is not associated with an increase in the risk of borderline tumors, its prolonged use increases this risk (57). In a case-control study, researchers concluded that, regardless of treatment, the risk of borderline ovarian tumors is two times higher in nulliparous women (58). Other researchers, however, could not show such a relationship (59, 60). A case-control study examined the correlation between five groups of fertility drugs including clomiphene citrate, human menopausal gonadotropins and follicle stimulating hormone, gonadotropin-releasing hormone analogs, human chorionic gonadotropins, progesterone and borderline ovarian tumors. The study found that use of progesterone was associated with an increased in borderline ovarian tumors, especially serous tumors, but no correlation was found between the borderline ovarian tumors and the use of other drugs (59). There is an ongoing debate about the relationship between fertility treatments and the risk of ovarian cancer and, so far, there has been no definitive evidence to confirm such relationship (24, 61, 62). The relationship between these drugs and the borderline ovarian tumors has only been reported in some studies (56, 63, 64).

#### Endometrial and Uterine Cancer

Endometrial cancer is one of the hormone-related cancers. Although the relationship between endometrial cancer and ovulation-inducting drugs is not clear, it appears that these drugs increase mitosis activity, DNA replication, mutation and malignancy by increasing serum level of estradiol during the follicular phase (65). However, by increasing oocyte cycles and pregnancy, the level of progesterone is significantly increased, and this plays a protective role against endometrial cancer. Results of several cohort studies showed an increase in endometrial cancer among women who used clomiphene citrate (24, 25). In a cohort study, researchers reported the highest increase in endometrial cancer among nulliparous women and those who have used more than 6 cycles of clomiphene (25). A cohort study which lasted for 30-years revealed that infertility is associated with an increased risk of endometrial cancer, and this risk does increase by ovulation induction (24). This is consistent with the finding of Brinton's study (66). The results of a case- control study supported previous research and stated duration of use of fertility drugs was positively associated with endometrial cancer risk (OR = 6.10; 95% CI, 0.96–38.6) (67). There have been a few studies that did not show any relationship between fertility treatment and endometrial cancer (68).

According to a cohort of 29,700 IVF women, the incidence of uterine sarcoma increases in women who have history of infertility (8). In a large cohort study, authors reported that the risk of uterine cancer in people taking ovulation-stimulating drugs is increased by 3 times, and this risk will increase by 8 times after taking clomiphene citrate for 12 months (29). Based on the results of a retrospective cohort study, the risk of uterine cancer increases with the increase in the dose of clomiphene citrate, its cycle of use, and the time since its first use, The risk is also increased in nulligravid and obese individuals (69). A cohort study concluded that consumption of more than 2,250 mg of clomiphene is associated with a 2.6-fold increase in the risk of uterus cancers (48). In 2009, researchers in a population based cohort study pointed to the relationship between the high dosages of gonadotropins and hCG and uterine cancer among the gonadotropins and hCG users (70). Infertility drugs may increase estrogen level during the follicular phase of ovulation stimulation cycles, and use of hCG and clomiphene for more than 6 months may increase the risk of uterine cancer (54). In general, uterine and endometrial cancers do not have high prevalence among different age groups, especially young people who are the target group in most studies, and therefore it is challenging to determine the relationship between endometrial cancer and the use of ovulation-inducing drugs.

#### Thyroid Cancer

The effect of exogenous hormonal agents on estrogen dependent malignancies has been discussed by many researchers. Stimulation of ovulation is associated with increased level of TSH hormone in the circulation, which causes cell proliferation in the gland (25). Some medications, such as clomiphene citrate, may have a greater effect on the thyroid gland due to longer half-life (71). The results of various studies suggest a link between thyroid cancer and ART (25, 71). A cohort study concluded that, the use of clomiphene citrate is associated with a 2-fold increase in the risk of thyroid cancer, and this risk is not statistically significant among the users of gonadotropins, hCG, and GnRH (71). The risk of thyroid cancer is higher among nulligravid women who take clomiphene citrate (72, 73). However, in a cohort study, the risk of thyroid cancer was higher among parous individuals who were taking clomiphene citrate (71). The use of clomiphene (RR = 2.28; 95% CI: 1.08–4.82) and progesterone (RR = 10.14; 95% CI: 1.93–53.33) is associated with an increased risk of thyroid cancer (71). Other studies have not shown any relationship between fertility drugs and thyroid cancer. Authors in a cohort study stated that there is no evidence of any relationship between gonadotropins and thyroid cancer (73). In a study, aggressive pattern of papillary thyroid carcinoma was seen among thyroid cancer patients who had received IVF treatment in the past and this may cause a delay in the thyroid cancer diagnosis. In this study, thyroid cancer was diagnosed after 4 years from the last IVF treatment (74). The present study did not find any relationship between fertility treatment and thyroid cancer.

#### Skin Cancer

Skin cancer endangers many people's lives due to death or disability. Age, gender, diet, and genetics affect incidence of this cancer (75). Potential effect of oral contraceptive pills on melanoma (76, 77), has been a source of concern for researchers about the role of fertility drugs on this cancer. In a cohort study, the incident of skin cancer (except melanoma) was higher in those who were using IVF treatment (78). Researchers stated that, although in general infertility treatment is not associated with the risk of malignant melanoma, the use of clomiphene citrate is associated with an increased risk of malignant melanoma, and this risk is more pronounced among those who have been waiting for pregnancy for more than a year (29). In a cohort study, authors concluded that although infertility is not associated with an increased risk of malignant melanoma, exposure to fertility drugs in women with infertile partners increases the risk of melanoma, and this risk is also reduced in women who receive a low dose of fertility drugs (79). According to a retrospective cohort study, infertile women with progesterone deficiencies had higher melanoma risk (80). In a retrospective cohort study among 8,422 women in 1965–1988, the researchers confirmed this result and concluded that, the use of fertility treatment does not significantly alter the risk of melanoma. In this study, clomiphene citrate had a stronger effect on the risk of melanoma (RR = 2; 95% CI:0.9–4.6) (73). Overall, the results of studies have shown no relationship between the risk of melanoma and fertility drugs.

#### Colon Cancer

Although lifestyle is one of the most important risk factors for colon cancer, sex hormones may also affect the etiology of colon cancer. The results of a cohort study showed that infertility treatment does not increase the risk of colon cancer (73). The result of a 21-year follow-up in a cohort study showed that ovarian stimulation for IVF does not increase the risk of breast cancer in comparison with the general population (81). Furthermore, after a 30 year follow-up of 9,892 women treated with pre- IVF fertility drugs, researchers found that these drugs have no effect on colorectal cancer. The increase in IVF cycles and a greater amount of gonadotropin ampoules did not alter this risk (72). Other studies showed that ovulation stimulation for IVF does not increase the risk of colon cancer in comparison with the general population (48, 81). Estrogen is considered to be protective against colorectal cancer (82), and so this cancer is expected to decrease in women who use fertility drugs. In addition, lower level of insulin- like growth factor I in women who use exogenous hormones, may play a role in reducing colorectal cancer in these group (81). Generally, the results of present study suggest that fertility treatment is not a risk factor for colon cancer.

#### Cervical Cancer

Cervical cancer is the fourth most common cancer among women (83). Twenty years follow up of 8,422 women during 1965–1988 showed that infertility treatment does not change the risk of cervical cancer (73). According to a cohort study, ovulation induction reduces the risk of cervical cancer (48). Other cohort studies reported a lower risk of cervical cancer in people undergoing IVF treatment (23, 78, 84). Ultimately, researchers have reported that, due to regular screening and periodic examinations of infertile people, the risk of cervical cancer is lower among these people (25). In addition, parity and full- term pregnancy increase the risk of cervical cancer, therefore, it seems that this cancer is less prevalent among infertile women (83).

### Non-Hodgkin's Lymphoma

Ovulation induction is associated with an increased risk of non-Hodgkin's lymphoma, and this risk is more pronounced in primiparous women as well as the first 5 years after childbirth (HR 2.63, 95% CI: 1.02, 6.82) (29).

## Conclusion

The purpose of this study was to determine the relationship between the use of fertility drugs and cancer. Due to the correlation between hormonal and reproductive factors and women's cancers, much more attention has been paid to the relationship between the use of fertility drugs and cancer in recent years. Although this relationship is theoretically justifiable, the complex and multiple factors that are involved in the onset of cancer make it difficult to determine the definite relationship between the use of these drugs and cancer. In spite of the relationship that exists between the ovulation-inducing drugs and cancer in some cohort studies, the results of our study showed no significant increase in the incidence of cancer by infertility treatment. According to the results of this study, there is no definitive relationship between the use of fertility drugs and cancer, and only some observational studies have pointed to this relationship. So, the following question still cannot be answered: Are fertility drugs safe?

Although, the small sample size, short-term follow-up, and lack of control over confounding variables are some of the most important limitations of such studies, more studies are needed to achieve a better result. Since, the large proportion of people who use ovulation-inducing drugs are young, long-term follow-up can better detect the onset of cancer among them. At the end, the relationship between infertility treatment and cancer incidence remains an open question.

## Author Contributions

All authors listed have made a substantial, direct and intellectual contribution to the work, and approved it for publication.

### Conflict of Interest Statement

The authors declare that the research was conducted in the absence of any commercial or financial relationships that could be construed as a potential conflict of interest.
